# A Five-Month-Old Boy With Hypotonia, Electrolyte Derangements, and Failure to Thrive

**DOI:** 10.7759/cureus.34226

**Published:** 2023-01-26

**Authors:** Samuel K Luketich, Seth J Deskins, Sydney Downey, Joseph Lynch

**Affiliations:** 1 Pediatrics, West Virginia University School of Medicine, Morgantown, USA; 2 Internal Medicine-Pediatrics, West Virginia University School of Medicine, Morgantown, USA

**Keywords:** aldosterone, nephrology, hypotonia, electrolyte disturbances, pediatrics

## Abstract

Failure to thrive in the setting of profound hypotonia and multiple electrolyte derangements is a challenging constellation of findings that offers a broad differential diagnosis for providers to consider. Initial management should focus on the stabilization of the patient and correction of potential life-threatening electrolyte derangements. Once completed, the diagnosis should be sought, and in this case, many were considered and ultimately ruled out with thorough history and physical examination. Laboratory abnormalities revealed the final diagnosis of pseudohypoaldosteronism and connected the case. With proper treatment, our patient had a resolution of laboratory anomalies along with improved growth and tone.

## Introduction

Failure to thrive is a commonly encountered problem in pediatrics. While most etiologies are inorganic, rarer causes can present. This is a case of the latter, where failure to thrive was the initial indication for hospital admission. History and physical exam revealed an infant with poor oral intake and profound hypotonia. Routine laboratory evaluation revealed multiple worrisome abnormalities. Further and more extensive workup showed the diagnosis. With electrolyte correction and treatment, we saw an improvement in intake, weight gain, and tone over time.

## Case presentation

**Figure 1:**
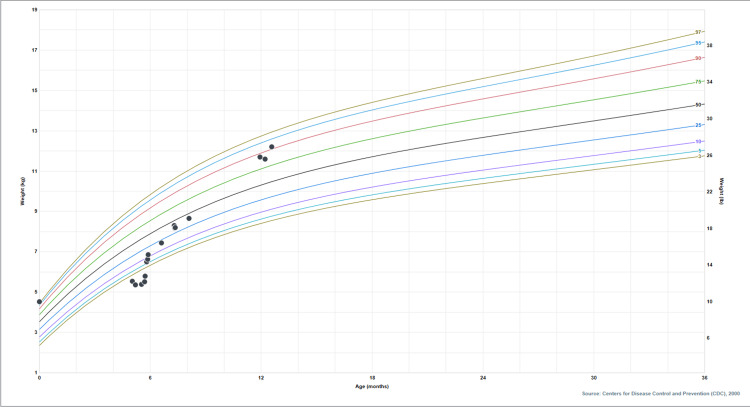
Growth chart

A previously healthy five-month-old male is admitted to our institution for failure to thrive (FTT), hypotonia, and metabolic derangements, including hyponatremia and hyperkalemia. He was born outside the United States, in Brazil, at 39 weeks, to non-consanguineous parents after a normal pregnancy and uncomplicated birth. Newborn screening was not performed. He is up to date on vaccinations. At initial presentation, his weight is 5.52 kg (<1st percentile), with length and head circumference being in the 60^th^ and 47^th^ percentiles, respectively. Birth weight is reported as 4.54 kg, and weight peaked at 6.35 kg two months before admission. The patient’s weight plateaued and then began to decrease. Figure 1 shows the growth chart.

This was caused by a poor suck on the bottle, vomiting, and worsening tone. The maternal breastmilk supply had diminished, and he was transitioned to formula feeding. Parents were syringe feeding before admission. His pediatrician increased the caloric density of his formula to 28 kcal/oz as his weight decreased to 5.35 kg. Even with this, he continued to lose weight and have a further decreasing tone, prompting hospital admission for failure to thrive and hypotonia. 

At admission, vitals are significant for fever (38°C) but otherwise normal for age, blood pressure (103/63 mmHg), heart rate (157 beats/min), and respiratory rate (40 breaths/min). A physical exam reveals a tired, limp infant who remains alert. He has fat wasting of the trunk and upper extremities with notable sparing of the lower extremities, a scaphoid-appearing abdomen, and marked global hypotonia. He has appropriate head control for his age but has a very weak trunk and cannot push himself up from his stomach or sit unsupported. He has an unremarkable cardiac exam with no murmurs. His abdomen is soft and non-distended, with normoactive bowel sounds and no organomegaly. He has normally formed Tanner 1 male genitalia with bilaterally descended testes. 

Initial laboratory studies are remarkable for profound hyponatremia (117 mmol/L, normal: 136-145 mmol/L), hyperkalemia (6.1 mmol/L, normal: 3.5-5.1 mmol/L), and hypochloremia (88 mmol/L, normal: 96-111 mmol/L) with a blood urea nitrogen and creatinine of 17 and 0.46, respectively (normal 5-20 mg/dL and 0.20-0.45 mg/dL). He also has a leukocytosis with a neutrophilic predominance (25.6x103/µL, normal: 6.5-13.3x103/µL), thrombocytosis (1069x103/µL, normal: 244-529x103/µL) and an elevated c-reactive protein of 24.1 mg/L (normal <8 mg/L). Electrocardiogram exhibits peaked T waves, left bundle-branch block, and shortened QRS duration prompting rapid treatment of hyperkalemia. Other notable laboratory studies include elevated alanine aminotransferase (249 u/L, normal: 5-33 u/L) and aspartate aminotransferase (101 u/L, normal: 20-65 u/L). Screening for fetal infections (TORCH) is negative. 

Other laboratory work-up was performed and included normal 17-hydroxyprogesterone (20 ng/dL, normal: <147 ng/dL) and cortisol (10.5 µd/dL, normal: 7-15 µd/dL). However, elevated aldosterone (745 ng/dL, normal: 2-70 ng/dL) and renin activity (48.71 ng/mL/h, normal: 0.25-5.82 ng/mL/h) levels reveal the actual diagnosis.

Urine culture reveals an infection with greater than 100 thousand colonies of pan-sensitive *Escherichia Coli*. Renal ultrasound shows grade 4 hydronephrosis on the right, with grade 1 on the left, along with a non-distended urinary bladder with some intraluminal debris noted. A voiding cystourethrogram (VCUG) was not performed while inpatient due to urology recommendations. VCUG at three months follow-up was normal. Blood cultures drawn at admission are negative. Multiple subspecialists are consulted and involved in his care. Complete neuro-axial imaging with MRI brain, cervical, thoracic, and lumbosacral spine is performed after neurology recommendation due to his hypotonia, but imaging is unrevealing. 

## Discussion

The differential diagnosis for our patient is broad, with consideration given to multiple metabolic, endocrine, and neurologic causes based on profound electrolyte abnormalities and hypotonia with FTT. Given his findings of the scaphoid abdomen and urinary tract abnormalities in the context of failure to thrive, Eagle-Barrett syndrome, which is characterized by these abnormalities [1], is considered. More common infectious, genetic, and metabolic disorders are considered due to international birth and lack of newborn screening [2] but are ruled out with negative TORCH infection titers and state newborn screen. With the fat wasting present, Barraquer-Simons syndrome, which involves fat wasting of the face and upper body [3], is briefly considered. However, neither his clinical course nor his metabolic derangements fit the timeline for this diagnosis. Electrolyte abnormalities of hyponatremia and hyperkalemia provoke further evaluation of the adrenal axis and renal system for diagnoses such as congenital adrenal hyperplasia (CAH) or aldosterone insufficiency. Normal 17-hydroxyprogesterone and cortisol levels rule out classic CAH, as this level would be elevated secondary to 21-hydroxylase deficiency [4]. However, elevated aldosterone and renin activity levels reveal the actual diagnosis.

Elevated aldosterone and renin activity levels from lab testing indicate pseudohypoaldosteronism. Although hyponatremia and hyperkalemia are consistent with hypoaldosteronism, there are true genetic defects along with transient causes of this disorder that mimic a low aldosterone state. An untreated urinary tract infection (UTI) potentially could have caused significant long-standing hydronephrosis and subsequent aldosterone resistance, although typically, some underlying renal/collecting system abnormality is found [5]. Complete normalization of aldosterone (16 ng/dL, normal: 2-70 ng/dL) and stabilization of electrolytes post-treatment supports a transient state and not an underlying genetic abnormality. Therefore, a diagnosis of transient pseudohypoaldosteronism secondary to urinary tract infection is more likely than either type I or type II pseudohypoaldosteronism caused by a genetic defect.

Pseudohypoaldosteronism is a group of disorders in which the kidney does not respond appropriately to aldosterone. Features of these disorders often include hyponatremia and hyperkalemia, as seen in our patient, along with elevated aldosterone and metabolic acidosis [6]. Transient pseudohypoaldosteronism, or secondary pseudohypoaldosteronism, is a condition in which a patient has hyponatremia, hyperkalemia, and hyperaldosteronism that resolves following the correction of an underlying abnormality [7]. Urinary tract malformations and infections are often the cause of transient pseudohypoaldosteronism [8], and this is likely the case with our patient, who had grade 4 hydronephrosis and a UTI. A delay in diagnosis may be due to the atypical presentation of UTIs in infants. Without reported fevers, a urinalysis was never obtained as an outpatient, and infection had not been considered before admission. The mechanism of the defect leading to pseudohypoaldosteronism is resistance to aldosterone in the renal tubules [5]. Elevated aldosterone levels should increase sodium reabsorption and potassium excretion, and the aldosterone would be attenuated due to negative feedback. Because there is no response to this signal, there is the continued production of renin and, thus, aldosterone. Both immature renal tubules [8,9] and UTI-induced resistance at the tubules [10] are hypothesized as causes of pseudohypoaldosteronism. One study on transient pseudohypoaldosteronism secondary to urinary tract malformations found that >80% of cases occur in males, and >90% of all cases occur before six months of age [9]. These patients present with failure to thrive, and expected electrolyte abnormalities are discovered on further workup [11]. CAH is the primary mimic of the clinical picture, and therefore evaluation of serum cortisol and 17-hydroxyprogesterone is essential [12], both of which should be normal in pseudohypoaldosteronism and delineate the diagnoses. Other presenting symptoms can include hypotonia, lethargy, and decreased oral intake [13,14], as with our patient. It is thought that the profound hypotonia is caused by prolonged electrolyte derangements, and thus why improvement is seen after corrections are made. 

Other types of pseudohypoaldosteronism are due to genetic defects. Type I pseudohypoaldosteronism is subdivided into an autosomal dominant form, causing mutations to the renal mineralocorticoid receptors, and an autosomal recessive form, causing mutations to the epithelial sodium channel. The recessive form affects several organ systems throughout the body and therefore has more severe consequences [15]. Alternatively, type II hypoaldosteronism has normal aldosterone and renin activity levels but abnormal kinases causing an increase in chloride resorption at the sodium-chloride co-transporter in the distal nephron and a consequent decrease in potassium secretion [6]. 

Resistance to aldosterone can be life-threatening. The first-line therapy of pseudohypoaldosteronism is to correct life-threatening electrolyte abnormalities and volume resuscitation. Our patient’s infection and the suspected underlying cause were treated with ceftriaxone, and his hyponatremia was corrected. We targeted sodium correction at a rate of 0.5-1.0mmol/L/hour to avoid central pontine myelinolysis with the administration of normal saline. One review study found that 76.5% of transient pseudohypoaldosteronism cases were resolved with medical treatment alone [9]. In a patient without previously identified urinary tract malformations, further workup with imaging should be performed [12], as in our patient, who is found to have unilateral grade 4 hydronephrosis. When there is a significant concern for CAH, such as virilization in a female infant, corticosteroids are often given while awaiting hormone levels to return, especially if hypotension is present [8]. Repeating aldosterone levels following correction of the electrolytes and treatment of the UTI is important to differentiate transient pseudohypoaldosteronism from genetic causes. If aldosterone levels remain elevated following treatment of electrolyte abnormalities and underlying causes, microarray analysis should be performed for genetic testing of type I or II pseudohypoaldosteronism [16]. 

Our patient’s sodium was corrected until within normal limits. A 14-day course of antibiotics to treat the *E. coli* UTI is started with intravenous ceftriaxone with the transition to oral amoxicillin/clavulanate for completion. Clinically, our patient demonstrates improvement in feeding, weight, and tone with the correction of electrolytes and approximately 48 hours of antibiotic treatment. He has an average weight gain of 22.3 g/day during hospitalization. Antibiotics are continued upon discharge, along with serial sodium checks. Three months later, the patient has been doing well since discharge, is continuing to gain weight, and meeting developmental milestones with now normal tone. 

## Conclusions

Here we present a case of an infant admitted for failure to thrive and profound hypotonia who was found to have multiple electrolyte derangements ultimately caused by pseudohypoaldosteronism. Pseudohypoaldosteronism should be suspected in patients with hyponatremia, hyperkalemia, and elevation in aldosterone, especially in the setting of urinary tract infection or underlying abnormality. This can be caused by underlying genetic defects or be transient secondary to undiagnosed urinary tract infections or malformation. With a high index of suspicion, along with thorough history, physical, and laboratory workup, pseudohypoaldosteronism can be identified and treatment initiated.
